# Comparison of Infectious Bronchitis Virus (IBV) Pathogenesis and Host Responses in Young Male and Female Chickens

**DOI:** 10.3390/v15122285

**Published:** 2023-11-22

**Authors:** Ishara M. Isham, Reham M. Abd-Elsalam, Motamed E. Mahmoud, Shahnas M. Najimudeen, Hiruni A. Ranaweera, Ahmed Ali, Mohamed S. H. Hassan, Susan C. Cork, Ashish Gupta, Mohamed Faizal Abdul-Careem

**Affiliations:** 1 Health Research Innovation Center 2C53, Faculty of Veterinary Medicine, University of Calgary, 3330 Hospital Drive NW, Calgary, AB T2N 4N1, Canada; fathimaishara.muhamm@ucalgary.ca (I.M.I.); reham.abdelsalam1@ucalgary.ca (R.M.A.-E.); motamed.ali@ucalgary.ca (M.E.M.); fathimashahnas.moham@ucalgary.ca (S.M.N.); hiruni.ranaweera@ucalgary.ca (H.A.R.); ahmed.ali@ucalgary.ca (A.A.); msh.hassan@ucalgary.ca (M.S.H.H.); sccork@ucalgary.ca (S.C.C.); ashish.gupta1@ucalgary.ca (A.G.); 2 Faculty of Veterinary Medicine, Cairo University, Giza 12211, Egypt; 3 Department of Animal Husbandry, Faculty of Veterinary Medicine, Sohag University, Sohag 82524, Egypt; 4 Department of Pathology, Beni-Suef University, Beni Suef 62521, Egypt; 5 Department of Avian and Rabbit Medicine, Faculty of Veterinary Medicine, Assiut University, Assiut 71515, Egypt

**Keywords:** infectious bronchitis virus (IBV), male chicken, female chicken, pathogenesis, immune response

## Abstract

Infectious bronchitis virus (IBV) is an avian coronavirus that causes a disease in chickens known as infectious bronchitis (IB). The pathogenesis of IBV and the host immune responses against it depend on multiple factors such as the IBV variant, breed and age of the chicken, and the environment provided by the management. Since there is limited knowledge about the influence of the sex of chickens in the pathogenesis of IBV, in this study we aim to compare IBV pathogenesis and host immune responses in young male and female chickens. One-week-old specific pathogen-free (SPF) White Leghorn male and female chickens were infected with Canadian Delmarva (DMV)/1639 IBV variant at a dose of 1 × 10^6^ embryo infectious dose (EID)_50_ by the oculo-nasal route while maintaining uninfected controls, and these chickens were euthanized and sampled 4- and 11-days post-infection (dpi). No significant difference was observed between the infected male and female chickens in IBV shedding, IBV genome load in the trachea, lung, kidney, bursa of Fabricius (BF), thymus, spleen, and cecal tonsils (CT), and IBV-induced lesion in all the examined tissues at both 4 and 11 dpi. In addition, there was no significant difference in the percentage of IBV immune-positive area observed between the infected male and female chickens in all tissues except for the kidney, which expressed an increased level of IBV antigen in infected males compared with females at both 4 and 11 dpi. The percentage of B lymphocytes was not significantly different between infected male and female chickens in all the examined tissues. The percentage of CD8+ T cells was not significantly different between infected male and female chickens in all the examined tissues except in the trachea at 11 dpi, where female chickens had higher recruitment when compared with male chickens. Overall, although most of the findings of this study suggest that the sex of chickens does not play a significant role in the pathogenesis of IBV and the host immune response in young chickens, marginal differences in viral replication and host responses could be observed to indicate that IBV-induced infection in male chickens is more severe.

## 1. Introduction

Infectious bronchitis virus (IBV) is an avian gamma coronavirus that belongs to the order Nidovirales, family Coronoviridae, and subfamily Orthocoronavirinae, and it causes infectious bronchitis (IB) [1]. IBV was first reported in North Dakota, United States of America (USA), in young chickens [2]. Although both the chicken (*Gallus gallus*) and pheasant (*Phasianus* spp.) are reported to be natural hosts of IBV, clinical IBV infections are predominantly reported in chickens [3]. IB is common and has a high morbidity; hence, it is one of the most frequently reported diseases in chickens. Furthermore, reports state that IB is second only to highly pathogenic avian influenza (HPAI) virus infection in terms of poultry diseases causing a significant impact on the global economy [4]. HPAI may be more frequently reported as it is a regulated and reportable disease. In addition, the emergence of new strains of IB and the failure of current vaccines to provide efficient and effective cross-protection to these new strains has led to an increase in the occurrence of IB in poultry farms. Hence, IB is one of the major challenges faced by the poultry industry, and major steps are used to control the spread of IB in order to prevent any significant economic losses [5]. Therefore, a better understanding of the pathogenesis of IBV and the immune responses generated against IBV is essential to combat IB. The pathogenesis of IBV and the host’s immune responses against it depend on multiple factors such as the characteristics of the pathogen (IBV), host (chicken), and environment.

The pathogenesis of IBV in chickens depends on the different variants of the virus, as different variants can lead to different tropisms of IBV and differing levels of severity of the disease. The gene coding for the spike (S)1 glycoprotein, which is the IBV receptor-binding domain (RBD), is a highly variable region in the IBV genome [6,7]. Hence, variations in the S1 protein can determine the virulence and tissue tropism of IBV as tissue tropism is determined by the avidity of the S1 protein to the α 2,3-linked sialic acid receptors in the host tissues [6,7]. The previous literature demonstrates that IBV is a multisystem disease that affects respiratory, reproductive, and renal tract tissues [8,9]. Some studies have also reported that IBV could infect the lymphoid organs of chickens [5,10,11]. In addition to the tropism of the virus, several comparative studies have demonstrated that the virulence of the virus differs between different variants within the respiratory [12,13], reproductive [14,15], and renal [16,17] systems. Studies have also described that the nutrition provided to chickens and their environmental conditions such as temperature, air quality, and light could also play some roles in the susceptibility of chickens to IBV infection [18,19].

Previously, it was shown that the age and breed of a chicken could play a role in the pathogenesis of IBV. Chickens of all ages are susceptible to IBV infection, but the severity of the disease is higher in younger chickens [20]. Studies report that with an increase in the age of chickens, the immune response and, thereby, the resistance to IBV infection and pathogenesis increases, hence resulting in lower IBV-induced nephropathic effects [16,20] and oviduct lesions [15]. However, another study by Macdonald and colleagues reported that younger chickens had a higher resistance to IBV than older chickens [21]. Another major factor of the host that could determine the pathogenesis of IBV is the breed of the chicken. Several experimental studies have demonstrated that the breed of the chicken could play a decisive role in susceptibility to IBV infection [18,22,23]. One such study reported that line 151 chickens are more susceptible to IBV M41 infection in comparison with line C White Leghorn chickens [23]. Another study reported that higher mortality was observed in IBV-infected broilers compared with layers [9]. 

A few studies have reported that the sex of a chicken could play a role in susceptibility to Arkansas (Ark), Australian T, and Australian N1/88 strains of IBV infection. A report by Cumming in 1969 stated that the susceptibility of male chickens is two-fold higher than that of female chickens [18]. In addition, a study in broiler chickens disclosed that female chickens developed immune responses faster than males by measuring the production of antibodies using a hemagglutination-inhibition assay and by evaluating antigen-specific T-lymphocyte proliferation experiments. Hence, the authors predicted that this difference in the rate of immune response development could be the leading cause of higher susceptibility to IBV infection in males [24]. Their study further stated that the mortality rate in male chickens was significantly higher than that of female chickens [24]. In another study on broiler chickens, female chickens had a higher phagocytic activity compared with male chickens, indicating that the innate immune response in females is more efficient than that observed in males [25]. However, a study on 18-day-old White Leghorn chickens hatched from specific-pathogen-free (SPF) fertile eggs reported that there was no significant difference in Ark-type IBV pathogenesis between sexes by assessing the viral load in the trachea and cecal tonsil (CT) and by assessing tracheal histomorphometry [26]. 

The existence of a relationship between steroid sex hormones and host immune response in humans, mice, and birds has been proclaimed [27,28,29]. It is widely suggested that sex hormones such as testosterone have an immunosuppressive effect, while estrogen has an immunoenhancing effect in hosts. A recent meta-analysis by Foo and colleagues in 2016 supported the past literature indicating that steroid sex hormones in males and females may affect immune responses in opposite directions, where testosterone in males resulted in an immunosuppressive effect while estrogen in females had an immunoenhancing effect [30]. Another review further reported that sex hormones could impact the strength of immune responses in males and females, where females demonstrated a stronger immune response than males [31]. In addition, a report on humans, rodents, and birds stated that estrogen stimulates humoral and cell-mediated immune responses in females, while testosterone has an opposite function in males [29]. Moreover, a study on broiler chicken demonstrated that the administration of estradiol 3-benzoate resulted in enhanced immune responses against *Escherichia coli* (*E. coli*) and sheep erythrocytes [24]. 

In recent years, a high incidence of the Delmarva (DMV)/1639 variant of IBV has been reported in layer flocks of Eastern Canada [32,33]. The DMV/1639 IBV causes cystic oviducts and false layer syndrome outbreaks in layers [34]. It is also responsible for a 30% drop in egg production in adult hens during the peak of lay [35]. Hence, it is imperative to study the factors that influence the pathogenesis of DMV/1639 in chickens. There is a scarcity of knowledge regarding the role of the sex of chickens in the pathogenesis of IBV infection and host immune response. Moreover, there are no studies comparing the pathogenicity of DMV/1639 IBV variants in young male and female chickens. Hence, in this study, we aimed to compare the IBV pathogenesis and host immune responses in young male and female chickens.

## 2. Materials and Methods

### 2.1. Virus

The Canadian IBV DMV/1639 strain (IBV/Ck/Can/17-036989) isolated from the kidney tissues of 22-week-old-infected chickens in a commercial layer flock in Eastern Canada was used as the challenge virus in this study [33]. The propagation of this viral strain was conducted by inoculating the allantoic cavities of 9-day-old SPF embryonated chicken eggs and harvesting the allantoic fluid 48 h post-inoculation after incubation (37.6 °C at 60% relative humidity from day 1 to 18 and 37.2 °C at 70% relative humidity from day 19 to 21). Following this, the virus was titrated, as previously described [34,35], where the 50% chicken embryo infectious dose (EID_50_) of the virus was calculated using the Reed and Muench method [36]. 

### 2.2. Experimental Animals

White Leghorn SPF one-day-old chickens were purchased from the Canadian Food Inspection Agency (CFIA), in Ottawa, Ontario, Canada, and they were sexed as described in Section 2.3. The male (number of chicken/*n* = 20) and female (*n* = 20) chickens were housed in two separate isolators at the Veterinary Science Research Station (VSRS) facility at the Spy Hill campus, University of Calgary. The chickens were allowed to adapt to the new environment for 6 days by providing feed and lighting according to the recommended management guidelines. The Veterinary Science Animal Care Committee (VSACC) of the University of Calgary provided ethical approval for this work (Protocol number: AC19-0011). 

### 2.3. Sex Determination in Chickens

The sex of the chickens was determined using a conventional polymerase chain reaction (PCR) assay, as previously described by He and colleagues, with some modifications [37]. Briefly, deoxyribonucleic acid (DNA) was extracted from the feather follicles of the chickens using a commercial DNA extraction kit (Qiagen, Hilden, North Rhine-Westphalia, Germany). The extracted DNA was amplified in a CFX 96-c1000 Thermocycler (Bio- Rad Laboratories, Mississauga, ON, Canada), where each reaction volume consisted of 2.5 µL 10X PCR buffer, 0.75 µL 50 mM MgCl_2_, 0.5 µL 10 mM dNTP, 0.5 µL 10 mM forward primer, 0.5 µL 10 mM reverse primer, and 0.1 µL of Taq DNA polymerase (ThermoFisher Scientific, Wilmington, DE, USA). The thermal profile for the amplification was, initial denaturation at 94 °C for 2 min; and 35 cycles of amplification with 30 s of denaturation at 94 °C, and 30 s of annealing at 55 °C and 30 s of elongation at 72 °C, followed by a final extension at 72 °C for 5 min. The forward and reverse primers used targeted the SWIM gene located on the W sexual chromosome, hence serving as a specific marker for females. The sequences of the forward and reverse primers were 5′GAGATCACGAACTCAACCAG-3′ and 5′CCAGACCTAATACGGTTTTACAG-3′, respectively [37]. Following the amplification of DNA, the PCR products were analyzed using agarose gel electrophoresis as described by He and colleagues [37].

### 2.4. Experimental Design

One-week-old male and female chickens were each randomly divided into two groups and housed in four separate isolators. One group of male and female chickens (*n* = 10 per each group) were challenged with 100 μL of the IBV DMV/1639 strain at a dose of 1 × 10^6^ EID_50_ by the oculo-nasal route, while the other group of male and female chickens were mock challenged with 100 μL of phosphate-buffered saline (PBS, Invitrogen Canada Inc., Burlington, ON, Canada) and maintained as uninfected controls. At 4 and 11 days post-infection (dpi), 1 mL of blood was collected from a randomly selected subset of chicken (*n* = 5) from the four experimental groups for the separation of serum. In addition, oropharyngeal (OP) and cloacal (CL) swabs were procured from the chicken from all the groups and were stored in 1 mL aliquots of cold PBS. Isoflurane anesthesia was performed on the chickens before euthanizing them by cervical dislocation. Subsequently, trachea, lung, kidney, bursa of Fabricius (BF), thymus, CTs, and spleen tissue samples were collected in ribonucleic acid (RNA) Save^®^ (Invitrogen Canada Inc., Burlington, ON, Canada) for extraction of RNA and in 10% neutral buffered formalin (VWR International, Edmonton, AB, Canada) for histopathology. The separated serum samples were stored at −20 °C, while the swab and tissue samples collected for RNA extraction were stored at −80 °C until further processing. The tissue samples fixed in 10% neutral buffered formalin were stored at room temperature.

### 2.5. Techniques

#### 2.5.1. Enzyme-Linked Immunosorbent Assay (ELISA)

The ELISA performed to quantify the serum anti-IBV antibody titer in chicken was performed with a commercial ELISA kit (IDEXX Laboratories, Inc., Westbrook, ME, USA). The ELISA was carried out according to the manufacturer’s instructions, and the antibody titers were calculated using the formula provided by the manufacturer. Accordingly, the titers above 396 (cut-off) were considered positive.

#### 2.5.2. RNA Extraction and cDNA Synthesis

The Trizol LS^®^ reagent (Invitrogen Canada Inc., Burlington, ON, Canada) and the Trizol reagent (Invitrogen Canada Inc., Burlington, ON, Canada) were used in accordance with the manufacturer’s instructions to extract RNA from swab and tissue samples, respectively. Then, the amount of extracted RNA was quantified at 260 nm wavelength with a Nanodrop 1000 spectrophotometer (ThermoFisher Scientific, Wilmington, DE, USA). Complementary DNA (cDNA) was synthesized using 1000 ng of RNA from swab samples and 2000 ng of RNA from tissue samples with the RT random primers high-capacity cDNA reverse transcriptase kit (Invitrogen Life Technologies, Carlsbad, CA, USA) following the manufacturer’s guidelines. 

#### 2.5.3. IBV Genome Load Quantification

The quantification of IBV genome load in swab and tissue cDNA samples was performed with quantitative PCR (qPCR) assay using Fast SYBR^®^ Green Master Mix (Quntabio^®^, Beverly, MA, USA). Each reaction volume incorporated 10 µL of SYBR Green master mix, 100 ng of interested cDNA sample, 0.5 µL 10 µM of forward primer, 0.5 µL 10 µM of reverse primer, and molecular biology-grade water to reach a final reaction volume of 20 µL. The qPCR assays were performed in a CFX 96-c1000 Thermocycler (Bio-Rad Laboratories, Mississauga, ON, Canada) with a thermal profile of 20 s of initial denaturation at 95 °C, 40 cycles of amplification with 3 s of denaturation at 95 °C, and 30 s of annealing at 60 °C. The forward and reverse primers used in this qPCR assay were 5′GACGGAGGACCTGATGGTAA-3′ and 5′CCCTTCTTCTGCTGATCCTG-3′, respectively, and they were targeted against the conserved IBV N gene [38].

#### 2.5.4. Immunohistochemistry (IHC)

IHC was performed on the paraffin sections of the tissue samples to quantify IBV nucleoprotein, CD8+ cells, and B cells. IHC was performed as previously described [10]. Briefly, the paraffin tissue sections on positively charged slides (VWR International, Radnor, PA, USA) were deparaffinized with xylene and then rehydrated in a descending serial concentration of alcohol. Subsequently, the endogenous peroxidase activity of the tissue specimens was blocked by incubating them in a 3% H_2_O_2_ solution in methanol for 10 min at room temperature. Following this, the viral epitopes in the tissue sections were unmasked by microwaving the sections at 850 W for 10–15 min with a 10 mM citrate buffer at pH 6.0. Then, the tissue sections were incubated overnight at 4 °C in a humidified chamber with the respective primary antibody. The mouse primary anti-IBV nucleoprotein antibody (Novus Biological, Bio-Techne, Toronto, ON, Canada) targeting the IBV antigen in cells, mouse anti-chicken CD8a antibody (SouthernBiotech, Birmingham, AL, USA) targeting the CD8 positive cells in tissues, and mouse anti-chicken Bu-1 antibody (SouthernBiotech, Birmingham, AL, USA) targeting the Bu-1 positive cells in tissues at dilutions of 1:400, 1:100, and 1:200 in PBS, respectively, were used for IHC staining. Succeeding this the tissue sections were incubated with goat anti-mouse IgG (H + L) secondary antibody (Vector Laboratories Inc., Newark, CA, USA) for 1 h at room temperature. Then, an ABC peroxidase kit and a 3,3′-Diaminobenzidine (DAB) substrate solution (Vector Laboratories, Newark, CA, USA) were used according to the manufacturer’s instructions to detect antibody binding. Washing of the tissue sections with Tris-buffered saline (TBS) for 5 min was performed following all the incubation steps described above. Then, the slides were counterstained with hematoxylin (Vector Laboratories, Newark, CA, USA) for 8 min, and bluing was accomplished by running the tissue sections under tap water for 30 min. Then, the tissue sections were dehydrated and cleaned with an ascending series of alcohol and xylene, respectively. The tissue sections were mounted in a toluene mounting solution (Vector Laboratories, Newark, CA, USA) and cover-slipped.

#### 2.5.5. Histopathology

The formalin-fixed tissue samples were sent to the Diagnostic Services Unit (DSU) at the University of Calgary, Faculty of Veterinary Medicine (UCVM), to prepare paraffin-embedded tissue blocks and hematoxylin–eosin (H&E)-stained tissue sections. The H&E stained tissue sections were examined under the light microscope (Olympus BX51, Center Valley, PA, USA) for histopathological lesions and scored as previously described with some modifications [39]. Each organ was scored based on several criteria, and the scoring system was performed as follows: normal (0), mild (1), moderate (2), and severe (3) for each criterion. Then, the total score was calculated for each organ per group. 

### 2.6. Statistical Analyses

The IBV genome load in swabs and tissues, percentage of IBV antigen, and histopathological lesion scores between the four groups were compared using the Kruskal–Wallis test followed by Dunn’s multiple comparisons test. The percentage of B (Bu-1+) and T (CD8+) lymphocytes in the tissues were compared with ordinary one-way ANOVA followed by Tukey’s multiple comparisons test. All the statistical analyses were performed with GraphPad Prism 9.2.0 Software (GraphPad Software, San Diego, CA, USA), and this software was also used to generate graphs. All the IHC images at 20× magnification stained for the IBV antigen were analyzed with Image J analyzer software version 1.46a (National Institute of Health, Bethesda, MD, USA). The immunostained B (Bu-1+) and T (CD8+) lymphocytes were counted at 20× magnification, and the percentage of positive cells was calculated relative to the total nuclei in each field using Fiji software as per the prior description [40]. For the analysis of all IHC images, five different fields from each tissue section were photographed and then analyzed.

## 3. Results

### 3.1. Clinical Manifestations

Only the DMV/1639-infected males showed clinical signs such as a mild increase in respiration at 4 dpi. Furthermore, the DMV1639-infected male or female birds did not reach the endpoints in the DMV/1639-infected male and female chickens during the period of this experiment.

### 3.2. Anti-IBV Antibody Titer

In all four experimental groups, the anti-IBV antibody titers in the serum were observed to be within the negative range (<396) at both 4 and 11 dpi. 

### 3.3. Viral Shedding

**Figure 1:**
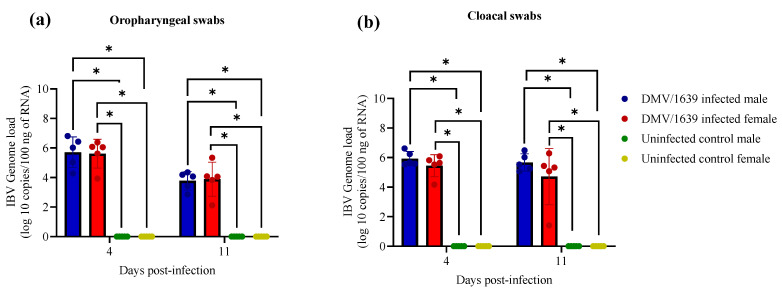
IBV genome load in (**a**) OP and (**b**) CL swabs at 4 and 11 dpi following infection with the Canadian DMV/1639 strain (IBV/Ck/Can/17–036989) of IBV. The average starting IBV genome load was quantified per 100 ng of the extracted RNA, and a comparison between groups was performed using the Kruskal–Wallis test followed by Dunn’s multiple comparisons test. The error bars represent the standard deviation (SD). Statistical significance: * *p* < 0.05.

The IBV genome was not detected in OP and CL swabs from uninfected male and female chickens at 4 and 11 dpi, while it was detected in the IBV-challenged chickens (Figure 1). A statistically significant difference in IBV genome load was not observed between the infected male and female chickens in the viral shedding via OP and CL swabs at 4 and 11 dpi (Figure 1, *p* > 0.05). 

### 3.4. IBV Genome Loads in Tissues

**Figure 2:**
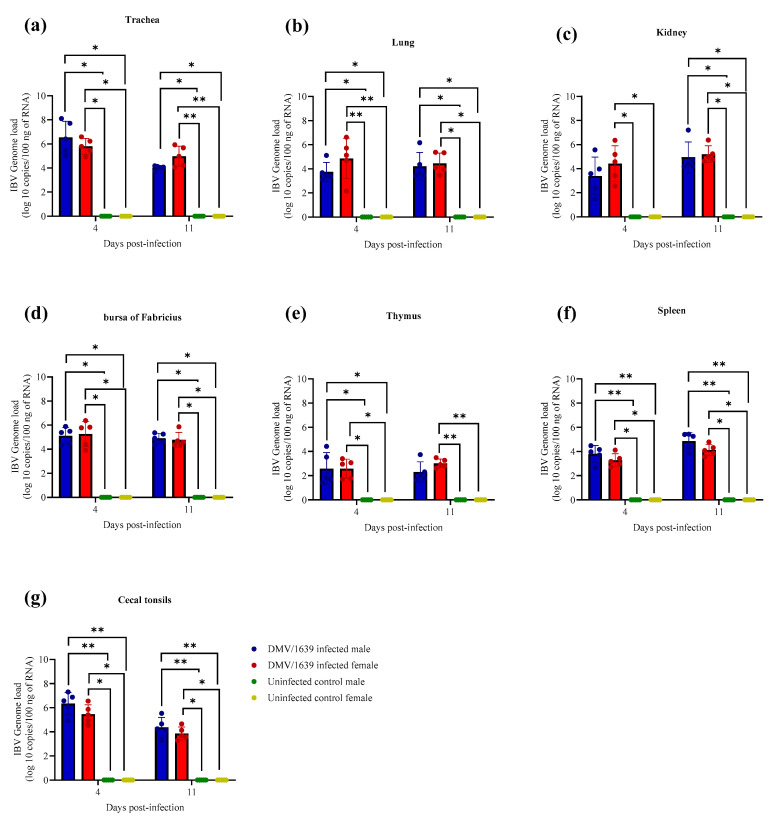
IBV genome loads in (**a**) trachea, (**b**) lung, (**c**) kidney, (**d**) bursa of Fabricius, (**e**) thymus, (**f**) spleen, and (**g**) cecal tonsils at 4 and 11 dpi following challenge with the Canadian DMV/1639 strain of IBV. The average starting IBV genome load was quantified per 100 ng of the extracted RNA, and a comparison between groups was performed using the Kruskal–Wallis test followed by Dunn’s multiple comparisons test. The error bars represent the SD. Statistical significance: * *p* < 0.05, ** *p* < 0.01.

The IBV genome was not detected with qPCR assays in all target organs of the uninfected male and female control chickens at both 4 and 11 dpi, thus stipulating no other source of IBV challenge (Figure 2). In addition, a significant difference in quantified IBV genome load was not demonstrated in all the targeted organs of the DMV/1639-infected male and female chickens at 4 and 11 dpi (Figure 2, *p* > 0.05).

### 3.5. IBV Antigen in Tissues

**Figure 3:**
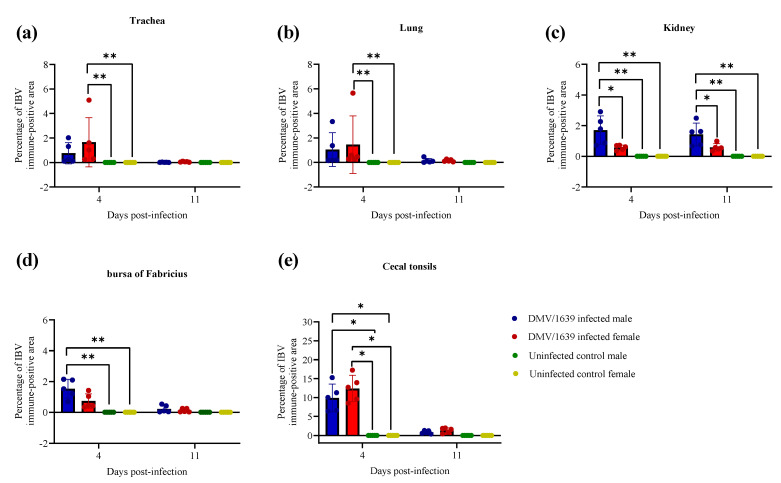
Percentage of IBV immune-positive area for IBV nucleoprotein in the (**a**) trachea, (**b**) lung, (**c**) kidney, (**d**) bursa of Fabricius, and (**e**) cecal tonsils at 4 and 11 dpi following challenge with the Canadian DMV/1639 strain of IBV. The comparison between groups was performed using the Kruskal–Wallis test followed by the Dunn’s multiple comparisons test, and the error bars represent the SD. Statistical significance: * *p* < 0.05 and ** *p* < 0.01.

**Figure 4:**
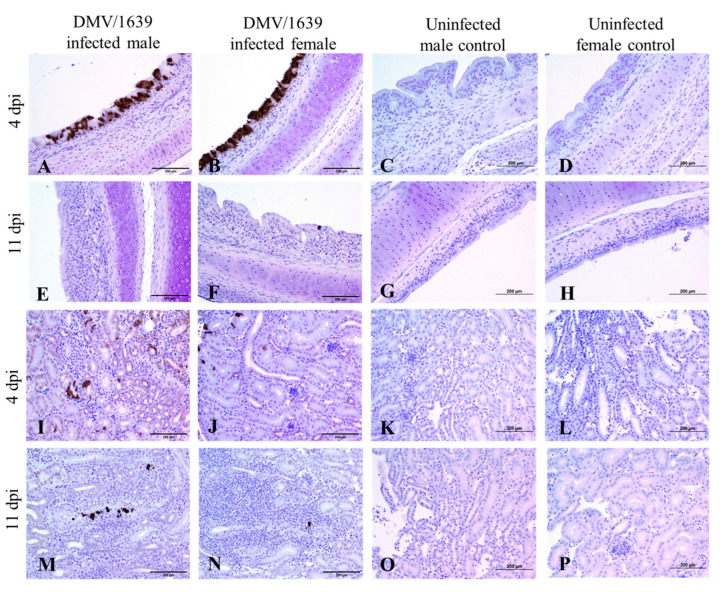
Photomicrograph of IBV nucleoprotein in the trachea and kidney. (**A**–**D**) Trachea of IBV DMV/1639-infected male, IBV DMV/1639-infected female, uninfected control male, and uninfected control female chickens at 4 dpi, respectively. (**E**–**H**) Trachea of IBV DMV/1639-infected male, IBV DMV/1639-infected female, uninfected control male, and uninfected control female chickens at 11 dpi, respectively. (**I**–**L**) Kidney of IBV DMV/1639-infected male, IBV DMV/1639-infected female, uninfected control male, and uninfected control female chickens at 4 dpi, respectively. (**M**–**P**) Kidney of IBV DMV/1639-infected male, IBV DMV/1639-infected female, uninfected control male, and uninfected control female chickens at 11 dpi, respectively.

**Figure 5:**
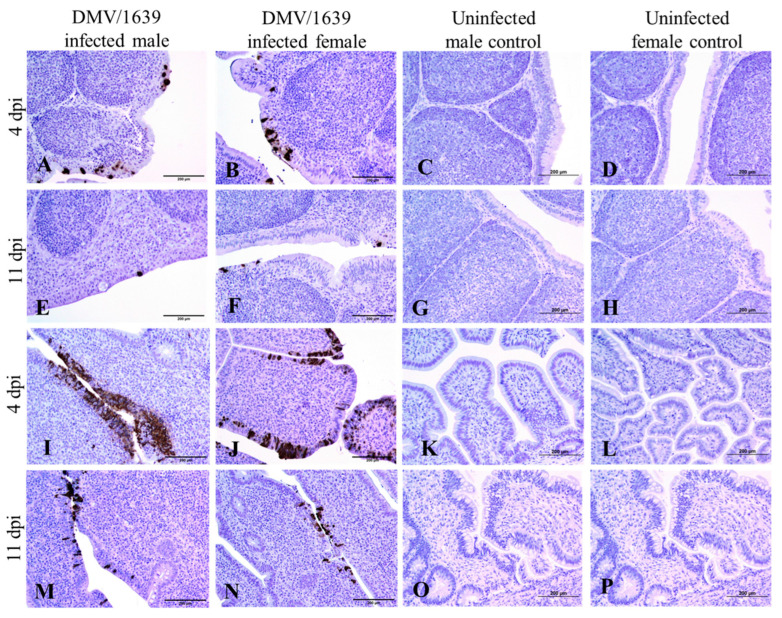
Photomicrograph of IBV nucleoprotein in the bursa of Fabricius and cecal tonsils. (**A**–**D**) bursa of Fabricius of IBV DMV/1639-infected male, IBV DMV/1639-infected female, uninfected control male, and uninfected control female chickens at 4 dpi, respectively. (**E**–**H**) bursa of Fabricius of IBV DMV/1639-infected male, IBV DMV/1639-infected female, uninfected control male, and uninfected control female chickens at 11 dpi, respectively. (**I**–**L**) Cecal tonsils of IBV DMV/1639-infected male, IBV DMV/1639-infected female, uninfected control male, and uninfected control female chickens at 4 dpi, respectively. (**M**–**P**) Cecal tonsils of IBV DMV/1639-infected male, IBV DMV/1639-infected female, uninfected control male, and uninfected control female chickens at 11 dpi, respectively.

**Figure 6:**
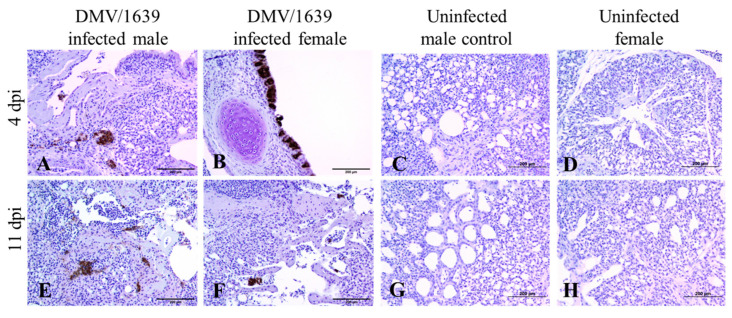
Photomicrograph of IBV nucleoprotein in the lung. (**A**–**D**) The lungs of IBV DMV/1639-infected male, IBV DMV/1639-infected female, uninfected control male, and uninfected control female chickens at 4 dpi, respectively. (**E**–**H**) The lungs of IBV DMV/1639-infected male, IBV DMV/1639-infected female, uninfected control male, and uninfected control female chickens at 11 dpi, respectively. The 4 dpi lung section of a female DMV1639-infected chicken shows a primary bronchus within lung tissue.

IBV immune-positive staining was not observed in all the target organs of the uninfected male and female control chickens at both 4 and 11 dpi (Figure 3, Figure 4, Figure 5 and Figure 6). On the contrary, immune-positive staining of IBV nucleoprotein was observed in the trachea, lung, kidney, BF, and CTs of DMV/1639-infected male and female chickens at 4 and 11 dpi (Figure 3, Figure 4, Figure 5 and Figure 6). The cells exhibiting IBV nucleoprotein revealed intra-cytoplasmic, brown fine to coarse crumbs, and IBV immune-positive cells were mostly distributed in the epithelial lining of the mucosa, renal tubular epithelium, and macrophages. The percentage of IBV immune-positive areas in the kidney of infected male chickens was significantly higher than that in infected female chickens at 4 and 11 dpi (Figure 3, *p* < 0.05). However, no significant difference was detected in the percentage of IBV immune-positive areas of the trachea, lung, BF, and CTs between DMV/1639-infected male and female chickens at 4 and 11 dpi (Figure 3, *p* > 0.05). Moreover, immune-positive staining of IBV nucleoprotein was not observed in the spleen or thymus of DMV/1639-infected male and female chickens at 4 and 11 dpi. 

### 3.6. Histopathology

**Figure 7:**
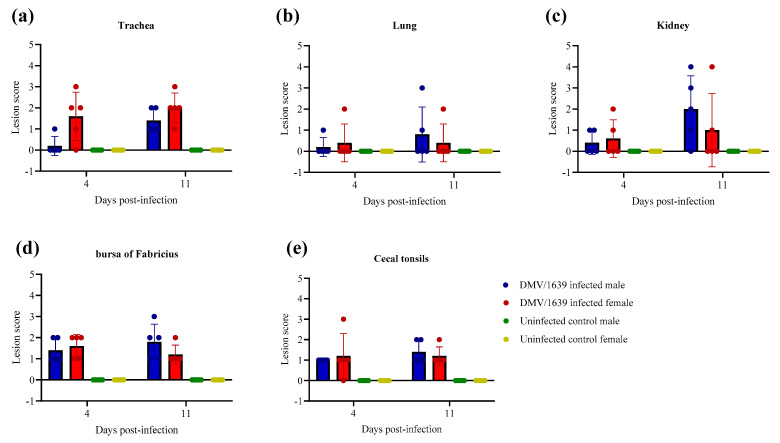
Lesion scores in the (**a**) trachea, (**b**) lung, (**c**) kidney, (**d**) bursa of Fabricius, and (**e**) cecal tonsils at 4 and 11 dpi following challenge with the Canadian IBV DMV/1639 strain. The comparison between groups was performed using the Kruskal–Wallis test followed by Dunn’s multiple comparisons test, and the error bars represent the SD.

The control uninfected male and female chickens at 4 and 11 dpi revealed normal histological architecture of the trachea, lung, kidney, thymus, spleen, BF, and CTs (Figure 7). IBV-induced lesions were observed in the trachea, lung, kidney, BF, and CTs of IBV-challenged male and female chickens, and there was no significant difference recorded in lesion scores between male and female IBV DMV/1639-infected chickens at 4 and 11 dpi (Figure 7, *p* > 0.05). IBV-induced lesions were not observed in the thymus or spleen of both IBV DMV/1639-infected male and female chickens at 4 and 11 dpi.

**Figure 8:**
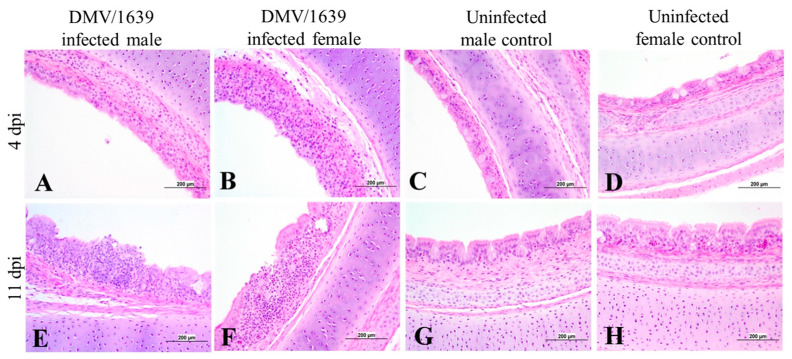
Photomicrographs of the trachea of (**A**) an IBV DMV/1639-infected male chicken at 4 dpi, (**B**) an IBV DMV/1639-infected female chicken at 4 dpi showing loss of cilia, degeneration, and necrosis of the epithelial lining, and mucosal and submucosal inflammatory cell infiltration, (**C**) an uninfected control male chicken at 4 dpi, and (**D**) an uninfected control female chicken at 4 dpi showing a normal histological picture, (**E**) an IBV DMV/1639-infected male at 11 dpi showing hyperplasia of the epithelial lining and focal area of epithelial necrosis with mucosal mononuclear cell aggregation, (**F**) an IBV DMV/1639-infected female chicken at 11 dpi showing focal epithelial loss with mucosal mononuclear inflammatory cell infiltration, (**G**) an uninfected control male chicken at 11 dpi, and (**H**) an uninfected control female chicken at 11 dpi.

The male and female IBV DMV/1639-infected chickens showed mild to moderate tracheal lesions at 4 dpi in the form of loss of cilia, degeneration and necrosis of columnar epithelium, mucus cell gland depletion, and lymphocytic infiltration in the mucosa and submucosa (Figure 8A,B). While at 11 dpi, the trachea showed focal areas of epithelium loss with alternated areas of epithelium and mucus cell gland hyperplasia and mononuclear cell infiltration in the mucosa and submucosa, leading to an increase in tracheal thickness (Figure 8E,F).

**Figure 9:**
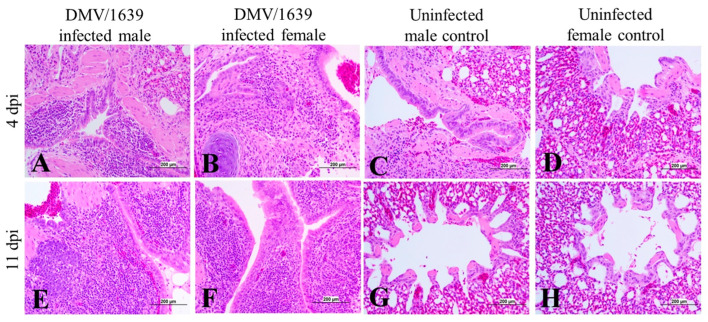
Photomicrographs of the lung of (**A**) IBV DMV/1639-infected male chicken at 4 dpi, (**B**) IBV DMV/1639-infected female chicken at 4 dpi showing mild bronchitis, (**C**) an uninfected control male chicken at 4 dpi, (**D**) an uninfected control female chicken at 4 dpi showing a normal histological picture, (**E**) an IBV DMV/1639-infected male chicken at 11 dpi, (**F**) an IBV DMV/1639-infected female chicken at 11 dpi showing moderate bronchitis, (**G**) an uninfected control male chicken at 11 dpi, and (**H**) an uninfected control female chicken at 11 dpi.

The histopathological changes in the lungs of both male and female infected chickens were minimal at 4 dpi with more severe lesions at 11 dpi (Figure 9A,B,E,F). Moderate bronchitis was detected in two male chickens and one female chicken in the IBV DMV/1639-infected groups at 11 dpi (Figure 9E,F).

**Figure 10:**
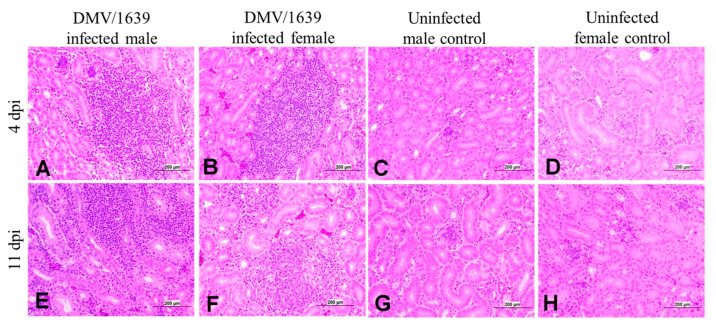
Photomicrographs of the kidney of (**A**) an IBV DMV/1639-infected male chicken at 4 dpi, (**B**) an IBV DMV/1639-infected female chicken at 4 dpi showing focal interstitial nephritis, (**C**) an uninfected control male chicken at 4 dpi, (**D**) an uninfected control female chicken at 4 dpi showing a normal histological architecture, (**E**) an IBV DMV/1639-infected male chicken at 11 dpi, (**F**) an IBV DMV/1639-infected female chicken at 11 dpi showing diffuse lymphoplasmocytic interstitial nephritis, (**G**) an uninfected control male chicken at 11 dpi, and (**H**) uninfected control female chicken at 11 dpi.

The male and female infected chickens exhibited mild renal tubular degeneration with focal aggregation of lymphoplasmocytes in the interstitial tissue of the kidney at 4 dpi (Figure 10A,B). However, moderate to severe lymphoplasmocytic interstitial nephritis was observed in both male and female infected chickens at 11 dpi (Figure 10E,F).

**Figure 11:**
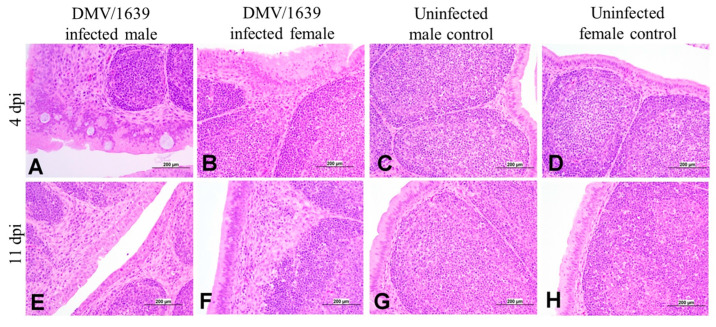
Photomicrographs of the bursa of Fabricius of (**A**) an IBV DMV/1639-infected male chicken at 4 dpi showing severe hyperplasia of lining epithelium with squamous cell metaplasia with ballooning and degeneration of some cells, (**B**) an IBV DMV/1639-infected female chicken at 4 dpi showing hyperplasia and squamous cell metaplasia of the plical lining epithelium, (**C**) an uninfected control male chicken at 4 dpi, (**D**) an uninfected control female chicken at 4 dpi showing normal epithelial lining and lymphoid follicles, (**E**) an IBV DMV/1639 infected male chicken at 11 dpi, (**F**) an IBV DMV/1639 infected female chicken at 11 dpi showing marked expansion of sub-epithelial and interfollicular tissue with mononuclear inflammatory cells, (**G**) uninfected control male chicken at 11 dpi, and (**H**) an uninfected control female chicken at 11 dpi.

The BF of male and female IBV DMV/1639-infected chickens exhibited mild to moderate histopathological changes at 4 and 11 dpi (Figure 11A,B,E,F). Mild to moderate lining epithelial cells hyperplasia, with degeneration and necrosis of some cells, epithelial and subepithelial infiltration with heterophils, lymphocytes, and macrophages were recorded at 4 dpi (Figure 11A,B). Moreover, the expansion of interfollicular and subepithelial tissues with edema and mononuclear cells and mild lymphoid depletion were also recorded. The nature of the BF lesions at 11 dpi was less severe (Figure 11E,F).

**Figure 12:**
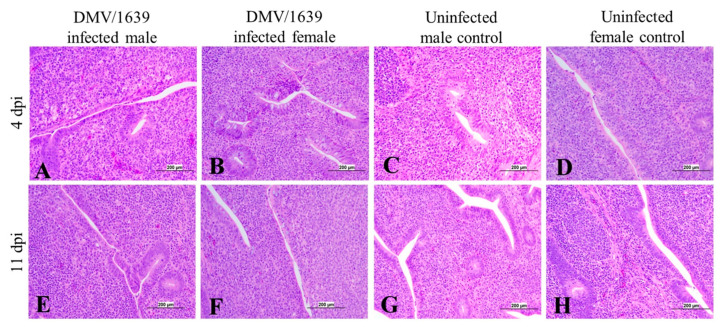
Photomicrograph of the cecal tonsils of (**A**) an IBV DMV/1639-infected male chicken at 4 dpi showing degeneration and necrosis of lining epithelium with vacuolation of sub-epithelial mononuclear cells, (**B**) an IBV DMV/1639-infected female chicken at 4 dpi showing mild degeneration of the epithelium, (**C**) an uninfected control male at 4 dpi, (**D**) an uninfected control female chicken at 4 dpi showing normal epithelial lining and lymphoid follicles, (**E**) an IBV DMV/1639-infected male chicken at 11 dpi, (**F**) an IBV DMV/1639 infected female chicken at 11 dpi show mild epithelial degeneration and necrosis, (**G**) an uninfected control male chicken at 11 dpi, and (**H**) an uninfected control female at 11 dpi.

The histopathological examination of the CTs of IBV DMV/1639-infected chickens revealed mild to moderate lymphoepithelial necrosis with sub-epithelial mononuclear cell aggregation, lymphoidal apoptosis, and lymphoidal depletion in interfollicular area at 4 and 11 dpi (Figure 12A,B,E,F).

### 3.7. Immunohistochemical Staining of B (BU-1+) and T (CD+8) Cells

**Figure 13:**
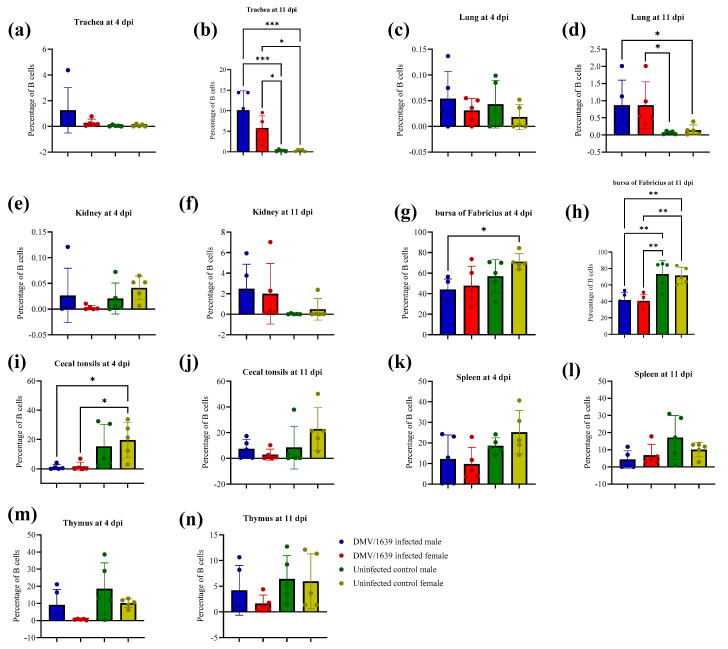
Percentage of B (BU-1+) cells in tissues infected with Canadian IBV DMV/1639 strain in male and female chickens and uninfected control male and female chickens at 4 and 11 dpi. (**a**,**b**) trachea, (**c**,**d**) lung, (**e**,**f**) kidney, (**g**,**h**) bursa of Fabricius, (**i**,**j**) cecal tonsils, (**k**,**l**) spleen and (**m**,**n**) thymus. The comparison between groups was performed using one-way ANOVA followed by Turkey’s multiple comparisons test, and the error bars represent the SD. Statistical significance: * *p* < 0.05, ** *p* < 0.01 and *** *p* < 0.001.

Figure 13 illustrates the percentage of B lymphocytes in the trachea, lung, kidney, CT, spleen, BF, and thymus of IBV-infected groups. No significant difference in B lymphocyte percentage was observed between the uninfected control male and female groups and the IBV-infected male and female groups in the trachea and lung at 4 dpi and in the kidney at both 4 and 11 dpi (Figure 13, *p* > 0.05). The percentage of B lymphocytes in the trachea and lung at 11 dpi was significantly higher in the IBV-infected male and female groups compared with the uninfected control male and female groups (Figure 13, *p* < 0.05). In addition, no significant difference was observed in the percentage of B lymphocytes in the trachea, lung, and kidney of IBV-infected male and female chickens (Figure 13, *p* > 0.05). In the BF, IBV-infected male and female chickens showed a significant reduction in the percentage of B lymphocytes compared with uninfected control male and female chickens (Figure 13, *p* < 0.01) at 11 dpi. However, a significant difference was not observed in the percentage of B lymphocytes in the BF of IBV-infected male and female chicken at 4 and 11 dpi (Figure 13, *p* > 0.05). Similarly, there was no significant difference in the percentage of B lymphocytes in the CT, spleen, and thymus between IBV-infected male and female chicken at both 4 and 11 dpi (Figure 13, *p* > 0.05). Figure S1 illustrates the distribution of B lymphocytes in all the examined tissues, and these B lymphocytes are stained brown and are indicated by arrows in the figures.

**Figure 14:**
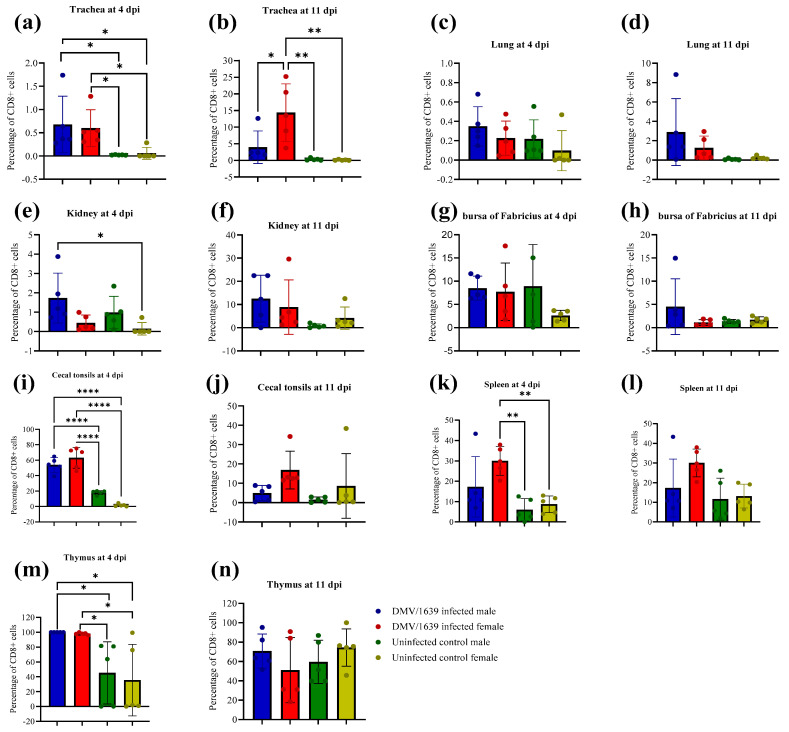
Percentage of T (CD8+) cells in tissues infected with the Canadian IBV DMV/1639 strain in male and female chickens and uninfected control male and female chickens at 4 and 11 dpi: (**a**,**b**) trachea, (**c**,**d**) lung, (**e**,**f**) kidney, (**g**,**h**) bursa of Fabricius, (**i**,**j**) cecal tonsils, (**k**,**l**) spleen, and (**m**,**n**) thymus. The comparison between groups was performed using one-way ANOVA followed by Turkey’s multiple comparisons test, and the error bars represent the SD. Statistical significance: * *p* < 0.05, ** *p* < 0.01, and **** *p* < 0.0001.

The percentages of T (CD8+) lymphocytes in the trachea, lung, kidney, BF, CTs, spleen, and thymus of the IBV-infected groups are demonstrated in Figure 14. A significant difference in the CD8+ T cell percentage was not discerned between the IBV-infected male and female groups and the uninfected control male and female groups in the lung and BF at both 4 and 11 dpi (Figure 14, *p* > 0.05). In addition, there was no statistically significant difference in the CD8+ T cell percentage in the IBV-infected male and female groups in comparison with the uninfected control male and female groups at 11 dpi in the kidney, CTs, spleen, and thymus (Figure 14, *p* > 0.05). Furthermore, a significantly higher percentage of CD8+ T cells were recorded in IBV-infected male and female chickens in comparison with uninfected control male and female chickens at 4 dpi in the trachea, CTs, and thymus (Figure 14, *p* < 0.05). The percentage of CD8+ T cells in IBV-infected female chickens was significantly higher in comparison with uninfected control male and female chickens at 4 dpi in the spleen, whilst the CD8+ T cell percentage in IBV-infected male was significantly higher than in the uninfected control female chickens at 4 dpi in the kidney (Figure 14, *p* < 0.05). However, there was no significant difference in the percentage of CD8+ T cells recorded between IBV-infected male and female chickens in all the tissues of interest at both 4 and 11 dpi except for the trachea (Figure 14, *p* > 0.05). The recruitment of CD8+ T cells in the IBV-infected female chickens was significantly higher than that in the IBV-infected male chickens in the trachea at 11 dpi (Figure 14, *p* < 0.05), whilst there was no significant difference in the levels of CD8+ T cells between IBV-infected male and female chickens in the trachea at 4 dpi (Figure 14, *p* > 0.05). Figure S2 shows the distribution of CD8+ T lymphocytes in all the examined tissues, and these CD8+ T lymphocytes are stained brown and are indicated by arrows in the figures.

## 4. Discussion

Due to its high rate of morbidity and challenges with disease control, IBV poses a significant economic threat to the poultry industry [4]. In addition, the prevalence of the DMV/1639 IBV strain in poultry farms in Eastern Canada has become alarmingly high [32,33]. Hence it is essential to understand the factors that govern the pathogenesis of the DMV/1639 IBV strain in chickens. Therefore, in this study, we aimed to determine if the sex of a chicken plays a role in the pathogenesis of DMV/1639 IBV and host immune responses in young male and female chickens. The results of this study were multifold. There was no significant difference in IBV genome load detected between DMV/1639 infected male and female chickens in viral shedding via OP and CL swabs and in tissue samples from all the targeted organs at both 4 and 11 dpi. Although no significant difference was detected in the percentage of IBV immune-positive areas of the trachea, lung, BF, and CTs between DMV/1639-infected male and female chickens at 4 and 11 dpi, the percentage of IBV immune-positive areas in the kidney tissues of infected male chickens was significantly higher than that of infected female chickens. In addition, no significant difference in IBV-induced lesion scores was detected between DMV/1639-infected male and female chicken at 4 and 11 dpi for all the targeted tissues. Furthermore, there was no significant difference in host response in terms of recruitment of B lymphocytes in all the tissues of interest at both 4 and 11 dpi. Moreover, the percentage of CD8+ T cells was not significantly different between infected male and female chickens in all the examined tissues except in the trachea at 11 dpi, where female chickens had higher recruitment of CD8+ T cells when compared with male chickens. 

Although there are several previous studies determining the impact that the IBV variant [12,13,14,15,16,17], age of chicken [16,20,21], and breed of chicken [9,18,22,23] have on the pathogenesis of IBV, there are very limited studies examining the role that the sex of a chicken plays in the pathogenesis of IBV and host immune response. In order to determine the role that the sex of a chicken plays in the pathogenesis of IBV and the subsequent immune response against the virus, we maintained all the other influencing factors at constant values. Hence, the breed and age of the chickens, the time of challenge, and the dose of IBV were kept the same between the infected male and female chickens. In addition, the condition of the isolators that housed these chickens and the feed and water were also maintained as uniformly as possible throughout the duration of the experiment for both male and female chickens. Furthermore, in order to minimize the variation in susceptibility to IBV, purebred White Leghorn chickens were used in this experiment instead of a crossbred chicken line, similar to a previous study aiming to investigate if sex influences the pathogenesis of IBV [26].

The anti-IBV antibody titers in the serum of all the experimental groups were within the negative range at both 4 and 11 dpi. The negative results in the IBV-infected male and female chickens at 4 dpi could be because the antibody-mediated immune response in chickens only starts at 4–5 dpi and the level of anti-IBV antibody in serum at this time will be very low [41,42]. In addition, the anti-IBV antibody titers in the serum of infected male and female chickens at 11 dpi were also negative. This could be because the birds in this study are very young, and hence, the amount of antibody produced by these birds will be low. ELISA has a comparatively lower assay sensitivity, and this could be a reason for obtaining negative results for infected chickens at 11 dpi [43]. In future studies, different commercial ELISA kits other than the kit used in this study could be used to quantify the anti-IBV antibody titers in the serum of young chickens. 

In terms of viral shedding, there was no statistically significant difference in the IBV genome load between the infected male and female chickens in both OP and CL swabs at 4 and 11 dpi. Although a few studies have reported a significant difference in IBV pathogenesis in the trachea of male and female chickens, these studies did not analyze viral shedding from the infected chickens [26,44]. Hence, this study provides some preliminary data comparing the IBV genome load in male and female chickens during the shedding of the virus. In addition, a significant difference was not observed in the IBV genome load between infected male and female chickens in the trachea, lung, kidney, BF, thymus, spleen, and CTs at both 4 and 11 dpi. In agreement, a previous study performed on 18-day-old White Leghorn chickens infected with the Ark type IBV variant also reported that there was no significant difference in the IBV genome load in the CTs between infected male and female chickens at 5 dpi [26]. However, in contrast to our finding, that study further reported that male chickens exhibited significantly higher viral loads in the trachea than that in females at 5 dpi [26]. This difference in the findings of the IBV genome load data in the two studies could be due to the difference in the IBV challenge strain (IBV DMV1639 vs. IBV Ark) and the dose used in the two studies.

To further determine the replication of IBV in the examined tissues, we stained the tissues for IBV nucleoprotein and analyzed the percentage of immune-positive areas with Image J software. In general, IBV antigen staining in the trachea was more intense than in the kidney tissues (Figure 4). In the trachea, lung, BF and CTs, a significant difference was not detected in the percentage of IBV immune-positive areas in DMV/1639-infected male and female chickens at 4 and 11 dpi. However, the percentage of IBV immune-positive areas in the kidney of infected male chickens was significantly higher than that in infected female chickens at 4 and 11 dpi. This lower percentage of IBV antigen in females could be due to the ability of females to prevent initial IBV infection. However, further studies are necessary to conclude if males are more susceptible to IBV DMV/1639 infection in kidney tissues compared with females. Since there are no past studies comparing and quantifying the distribution of IBV antigen in tissues of infected male and female chickens, this study provides some important preliminary data to design future studies. In this study, we observed a discrepancy in the detection of the IBV genome load and IBV antigen in the examined tissues. There was no significant difference in the IBV genome load between infected male and female chickens in all the examined tissues at both 4 and 11 dpi, while a higher expression of IBV antigen was observed in infected male chickens compared with the infected females in the kidney at 4 and 11 dpi. Furthermore, although the IBV genome was detected in all the examined tissues, the IBV antigen was not detected in the spleen or thymus of infected chickens. This discrepancy is not alarming since the qPCR technique detects both replicating and non-replicating IBV in tissues, while the immunohistochemistry technique detects only the IBV antigen expressed by the replicating IBV [45,46]. In addition, since the qPCR technique has a higher sensitivity compared with the immunohistochemistry technique, some tissues positive for the IBV viral genome could be negative for the IBV antigen [47,48]. 

Furthermore, histopathology was performed to compare IBV-induced lesions in the trachea, lung, kidney, BF, and CTs, and we observed no significant difference in the lesion scores between IBV DMV/1639-infected male and female chickens at 4 and 11 dpi. In agreement with these results, a previous study observing and comparing the tracheal histomorphometry in terms of mucosal thickness and lymphocyte infiltration in the trachea of IBV-infected male and female chickens reported that there was no significant difference in both the thickness of the tracheal mucosa and lymphocytic infiltration in trachea between the two sexes [26]. However, another study on 65-week-old hens and cockerels, challenged with T and N1/88 Australian IBV strains, reported that the cockerels had more severe tracheal lesions in terms of edema in mucosa and alveolar mucous gland hypertrophy compared with the hens [44]. That study further stated that there was no significant difference in lymphocytic infiltration in the trachea of the challenged hens and cockerels [44]. The contradiction between the results of that study and our study could be due to the difference in the age of chickens during the IBV challenge and the difference in the IBV strains used for the challenge (IBV DMV1639 vs IBV Australian T and N1/88). Moreover, similar to the observations of our study, that study also reported that no significant difference was observed in the histopathological lesions between male and female chickens in the kidneys [44].

The host immune response in chickens during an IBV infection is mediated by innate and adaptive immune responses. The adaptive immune response is essential to reduce and control an IBV infection in tissues, clearance of IBV, and the reduction in the shedding of IBV [49]. Several previous studies have reported the recruitment of lymphocytes in the target organs following IBV infection [13,35,50]. In this study, the recruitment of B and T lymphocytes in the target organs was examined and compared between IBV DMV/1639-infected male and female chickens. Recruitment of B lymphocytes in the trachea and lung was illustrated with the significant increase in the percentage of B cells at 11 dpi in IBV-infected male and female chickens compared with uninfected control male and female chickens. This could be indicative of the initiation of an adaptive immune response in chickens and the lymphocyte infiltration in infected tissues [51]. On the contrary, a significant decline was noted in the percentage of B cells in BF of IBV DMV/1639 infected male and female chickens compared with the uninfected controls at both 4 and 11 dpi. A prior study reported similar observations of depleted B cells in the BF of IBV-infected birds due to indirect follicular destruction in the BF [52]. However, no significant difference was discovered in the percentage of B cells at 4 and 11 dpi between IBV DMV/1639-infected male and female chickens in all the tissues of interest at both 4 and 11 dpi. These findings could indicate that there is no difference in the B cell response mounted by the chicken based on sex. Since there are no past studies comparing the recruitment of B cells in male and female chickens, the results of this study could be used as basic data to design future studies to investigate the difference in host immune response in male and female chickens. 

Cytotoxic T cells (CD8+ T-cells) are essential in eliminating IBV-infected cells during an infection [53]. The influx of CD8+ T cells at the site of IBV infection to control the replication and spread of the virus has been documented in the past literature [35,49,54]. A significantly higher percentage of CD8+ T cells were recorded in IBV-infected male and female chickens in comparison with uninfected control male and female chickens at 4 dpi in the trachea, CTs, and thymus in this study. These observations are in agreement with the findings reported in previous studies, which showed increased levels of recruitment of CD8+ T cells in the kidney of infected birds from 3 dpi onward [49,55]. Moreover, no significant difference was revealed in the percentage of CD8+ T cells between IBV-infected male and female chickens at both 4 and 11 dpi in all the examined tissues except for the trachea. This could suggest that no significant difference exists in the host immune response against IBV infection in most organs. Although there are no prior studies examining and comparing the recruitment of CD8+ T cells in IBV-infected male and female chickens, a study by Leitner and colleagues described that there was no significant difference in the T cells in peripheral blood lymphocytes in infected male and female chickens [24]. However, in our study, in the trachea of IBV-infected female chickens, the recruitment of CD8+ T cells was significantly higher than that in IBV-infected male chickens at 11 dpi. This observation is in support of some previous studies that reported that males are more susceptible to viral infection than females [18,24]. 

This study presented some evidence to indicate a difference in the pathogenesis of IBV DMV/1639 in male and female chickens due to the presence of increased levels of IBV antigen in the kidneys of infected male chickens compared with females at 4 and 11 dpi and in the host immune response due to the presence of comparatively higher levels of CD8+ T cell recruitment in the trachea of infected females compared with males at 11 dpi. However, these findings alone are not sufficient to suggest that chickens of different sexes respond differently to an IBV infection. Moreover, the vast majority of the findings of this study advocate that the sex of a chicken does not play a role in the pathogenesis of IBV or in the host immune response in young chickens up to 18 days of age. Further studies comparing the pathogenesis and immune response in chickens of different ages and breeds challenged with different doses and variants of IBV are essential to conclude if sex plays a role in influencing the pathogenesis of IBV and immune response against IBV in chickens. 

The difference in the IBV antigen and CD8+ T cells found in males and females must be investigated in the future to determine if male chickens are more susceptible to IBV DMV1639 infection compared with female chickens, as this could have implications for the transmission of IBV in poultry farms where both male and female chickens are housed together for the purpose of breeding. If males are more susceptible to IBV infection, they could facilitate the transmission of IB in commercial flocks when housed together in both layer and broiler chickens. Hence, an understanding of the gender difference in IBV susceptibility is imperative for the control of IBV in the poultry industry in Canada and globally. The vast majority of IBV genome load, IBV antigen, and histopathology data presented in this study, in addition to the B and T cell recruitment results, suggest that the sex of a chicken does not play an influential role in the pathogenesis of IBV and the host immune response against IBV infection in young chickens. These preliminary data could encourage researchers to use young male chickens for experimental purposes such as infection studies and vaccine efficacy studies instead of or with females and avoid euthanasia of male chickens. Reports from the Food and Agriculture Organization of the United Nations suggest that every year, around 6 billion day-old male chickens are euthanized all over the world as they are considered futile products [37]. This is raising ethical concerns in both the poultry and research industry. Hence, the use of young male chickens instead of or with females for suitable research regarding IBV tropism, IBV pathogenesis, and vaccination trials could be a viable solution to this ethical concern. However, in comparison with the 6 billion day-old male chickens euthanized every year, only a small number of male chickens could be used as experimental animals in studies. Therefore, the use of male chickens in experiments does not completely solve this ethical problem. Moreover, most experiments require adult chickens, and this study assessed a gender difference in IBV susceptibility in older chickens. Hence, future studies must be conducted to compare the pathogenicity of IBV and host immune response against IBV infection in adult male and female chickens. 

In conclusion, a marginal difference was observed between the IBV DMV/1639 infected male and female chickens in the viral replication and host responses and these observations agree with previous observations of susceptivity of males for IBV infection [24,44]. Further studies are necessary to elucidate if the increased severity of IBV infection in males is dependent on the infecting IBV strain.

## Data Availability

The datasets used and/or analyzed within the frame of this study will be provided by the corresponding author upon reasonable request.

## Supplementary Materials

The following supporting information can be downloaded at: https://www.mdpi.com/article/10.3390/v15122285/s1, Figure S1: Immunohistochemical detection of B (Bu-1+) lymphocytes in the trachea (A), lung (B), kidney (C), cecal tonsils (D), spleen (E), bursa of Fabricius (F), and thymus (G) collected at 4 and 11 dpi following infection with the Canadian IBV DMV/1639 strain. Bu-1+ cells were stained with deep brown (indicated with black arrows). Scale bar = 50 μm; Figure S2: Immunohistochemical detection of CD8+ cells in the trachea (H), lung (I), kidney (J), cecal tonsils (K), spleen (L), bursa of Fabricius (M), and thymus (N) collected 4- and 11-days following infection with the Canadian IBV DMV/1639 strain. CD8+ cells were stained with deep brown (indicated with black arrows). Scale bar = 50 μm.

## References

[1] Woo P.C.Y., de Groot R.J., Haagmans B., Lau S.K.P., Neuman B.W., Perlman S., Sola I., van der Hoek L., Wong A.C.P., Yeh S.H. (2023). ICTV Virus Taxonomy Profile: Coronaviridae 2023. J. Gen. Virol..

[2] Schalk A.F., Hawn M.C. (1931). An Apparently New Respiratory Disease of Baby Chicks. J. Am. Vet. Med. Assoc..

[3] Cavanagh D., Mawditt K., Welchman Dde B., Britton P., Gough R.E. (2002). Coronaviruses from pheasants (*Phasianus colchicus*) are genetically closely related to coronaviruses of domestic fowl (infectious bronchitis virus) and turkeys. Avian Pathol..

[4] TAFS-Forum (2011). World Livestock Disease Atlas a Quantitative Analysis of Global Animal Health Data (2006–2009).

[5] Ambali A.G., Jones R.C. (1990). Early pathogenesis in chicks of infection with an enterotropic strain of infectious bronchitis virus. Avian Dis..

[6] Barnard D.L. (2008). Coronaviruses: Molecular and Cellular Biology. Future Virol..

[7] Wickramasinghe I.N., de Vries R.P., Gröne A., de Haan C.A., Verheije M.H. (2011). Binding of avian coronavirus spike proteins to host factors reflects virus tropism and pathogenicity. J. Virol..

[8] Bande F., Arshad S.S., Omar A.R., Bejo M.H., Abubakar M.S., Abba Y. (2016). Pathogenesis and Diagnostic Approaches of Avian Infectious Bronchitis. Adv. Virol..

[9] Ignjatovic J. Epidemiology of infectious bronchitis in Australia. Proceedings of the 1st International Symposium on Infectious Bronchitis.

[10] Isham I.M., Hassan M.S.H., Abd-Elsalam R.M., Ranaweera H.A., Mahmoud M.E., Najimudeen S.M., Ghaffar A., Cork S.C., Gupta A., Abdul-Careem M.F. (2023). Impact of Maternal Antibodies on Infectious Bronchitis Virus (IBV) Infection in Primary and Secondary Lymphoid Organs of Chickens. Vaccines.

[11] Najimudeen S.M., Abd-Elsalam R.M., Ranaweera H.A., Isham I.M., Hassan M.S.H., Farooq M., Abdul-Careem M.F. (2023). Replication of infectious bronchitis virus (IBV) Delmarva (DMV)/1639 variant in primary and secondary lymphoid organs leads to immunosuppression in chickens. Virology.

[12] Cubillos A., Ulloa J., Cubillos V., Cook J.K. (1991). Characterisation of strains of infectious bronchitis virus isolated in Chile. Avian Pathol..

[13] Raj G.D., Jones R.C. (1996). An in vitro comparison of the virulence of seven strains of infectious bronchitis virus using tracheal and oviduct organ cultures. Avian Pathol..

[14] Cook J.K., Huggins M.B. (1986). Newly isolated serotypes of infectious bronchitis virus: Their role in disease. Avian Pathol..

[15] Crinion R.A., Hofstad M.S. (1972). Pathogenicity of four serotypes of avian infectious bronchitis virus for the oviduct of young chickens of various ages. Avian Dis..

[16] Albassam M.A., Winterfield R.W., Thacker H.L. (1986). Comparison of the nephropathogenicity of four strains of infectious bronchitis virus. Avian Dis..

[17] Chandra M. (1987). Comparative nephropathogenicity of different strains of infectious bronchitis virus in chickens. Poult Sci..

[18] Cumming R.B. (1969). The control of avian infectious bronchitis/nephrosis in Australia. Aust. Vet. J..

[19] Hofmann T., Schmucker S.S., Bessei W., Grashorn M., Stefanski V. (2020). Impact of Housing Environment on the Immune System in Chickens: A Review. Animals.

[20] Animas S.B., Otsuki K., Tsubokura M., Cook J.K. (1994). Comparison of the susceptibility of chicks of different ages to infection with nephrosis/nephritis-causing strain of infectious bronchitis virus. J. Vet. Med. Sci..

[21] Macdonald J.W., Randall C.J., McMartin D.A. (1980). An inverse age resistance of chicken kidneys to infectious bronchitis virus. Avian Pathol..

[22] Bumstead N., Huggins M.B., Cook J.K. (1989). Genetic differences in susceptibility to a mixture of avian infectious bronchitis virus and Escherichia coli. Br. Poult. Sci..

[23] Nakamura K., Cook J.K., Otsuki K., Huggins M.B., Frazier J.A. (1991). Comparative study of respiratory lesions in two chicken lines of different susceptibility infected with infectious bronchitis virus: Histology, ultrastructure and immunohistochemistry. Avian Pathol..

[24] Leitner G., Heller E.D., Friedman A. (1989). Sex-related differences in immune response and survival rate of broiler chickens. Vet Immunol. Immunopathol..

[25] Younis M.E.M., Jaber F.A., Majrashi K.A., Ghoneim H.A., Shukry M., Shafi M.E., Albaqami N.M., Abd El-Hack M.E., Abo Ghanima M.M. (2023). Impacts of synthetic androgen and estrogenic antagonist administration on growth performance, sex steroids hormones, and immune markers of male and female broilers. Poult. Sci..

[26] Lockyear O., Breedlove C., Joiner K., Toro H. (2022). Distribution of Infectious Bronchitis Virus Resistance in a Naïve Chicken Population. Avian Dis..

[27] Taneja V. (2018). Sex Hormones Determine Immune Response. Front. Immunol..

[28] Ansar Ahmed S., Penhale W.J., Talal N. (1985). Sex hormones, immune responses, and autoimmune diseases. Mechanisms of sex hormone action. Am. J. Pathol..

[29] Schuurs A.H., Verheul H.A. (1990). Effects of gender and sex steroids on the immune response. J. Steroid Biochem..

[30] Foo Y.Z., Nakagawa S., Rhodes G., Simmons L.W. (2017). The effects of sex hormones on immune function: A meta-analysis. Biol. Rev. Camb. Philos. Soc..

[31] Roved J., Westerdahl H., Hasselquist D. (2017). Sex differences in immune responses: Hormonal effects, antagonistic selection, and evolutionary consequences. Horm. Behav..

[32] Gagnon C.A., Bournival V., Koszegi M., Nantel-Fortier N., St-Sauveur V.G., Provost C., Lair S. (2022). Quebec: Avian pathogens identification and genomic characterization: 2021 annual review of the Molecular Diagnostic Laboratory, Université de Montréal. Can. Vet. J..

[33] Hassan M.S.H., Ojkic D., Coffin C.S., Cork S.C., van der Meer F., Abdul-Careem M.F. (2019). Delmarva (DMV/1639) Infectious Bronchitis Virus (IBV) Variants Isolated in Eastern Canada Show Evidence of Recombination. Viruses.

[34] Hassan M.S.H., Ali A., Buharideen S.M., Goldsmith D., Coffin C.S., Cork S.C., van der Meer F., Boulianne M., Abdul-Careem M.F. (2021). Pathogenicity of the Canadian Delmarva (DMV/1639) Infectious Bronchitis Virus (IBV) on Female Reproductive Tract of Chickens. Viruses.

[35] Hassan M.S.H., Buharideen S.M., Ali A., Najimudeen S.M., Goldsmith D., Coffin C.S., Cork S.C., van der Meer F., Abdul-Careem M.F. (2022). Efficacy of Commercial Infectious Bronchitis Vaccines against Canadian Delmarva (DMV/1639) Infectious Bronchitis Virus Infection in Layers. Vaccines.

[36] Reed L.J., Muench H. (1938). A simple method of estimating fifty per cent endpoints. Am. J. Epidemiol..

[37] He L., Martins P., Huguenin J., Van T.N., Manso T., Galindo T., Gregoire F., Catherinot L., Molina F., Espeut J. (2019). Simple, sensitive and robust chicken specific sexing assays, compliant with large scale analysis. PLoS ONE.

[38] Kameka A.M., Haddadi S., Kim D.S., Cork S.C., Abdul-Careem M.F. (2014). Induction of innate immune response following infectious bronchitis corona virus infection in the respiratory tract of chickens. Virology.

[39] Hussein E.A., Hair-Bejo M., Adamu L., Omar A.R., Arshad S.S., Awad E.A., Aini I. (2018). Scoring System for Lesions Induced by Different Strains of Newcastle Disease Virus in Chicken. Vet. Med. Int..

[40] Schindelin J., Arganda-Carreras I., Frise E., Kaynig V., Longair M., Pietzsch T., Preibisch S., Rueden C., Saalfeld S., Schmid B. (2012). Fiji: An open-source platform for biological-image analysis. Nat. Methods.

[41] Sego T.J., Aponte-Serrano J.O., Ferrari Gianlupi J., Heaps S.R., Breithaupt K., Brusch L., Crawshaw J., Osborne J.M., Quardokus E.M., Plemper R.K. (2020). A modular framework for multiscale, multicellular, spatiotemporal modeling of acute primary viral infection and immune response in epithelial tissues and its application to drug therapy timing and effectiveness. PLoS Comput. Biol..

[42] Yang W., Liu X., Wang X. (2023). The immune system of chicken and its response to H9N2 avian influenza virus. Vet. Q..

[43] Boonham N., Kreuze J., Winter S., van der Vlugt R., Bergervoet J., Tomlinson J., Mumford R. (2014). Methods in virus diagnostics: From ELISA to next generation sequencing. Virus Res..

[44] Chousalkar K.K., Roberts J.R., Reece R. (2007). Comparative histopathology of two serotypes of infectious bronchitis virus (T and n1/88) in laying hens and cockerels. Poult. Sci..

[45] Klein D. (2002). Quantification using real-time PCR technology: Applications and limitations. Trends Mol. Med..

[46] Eyzaguirre E., Haque A.K. (2008). Application of immunohistochemistry to infections. Arch. Pathol. Lab. Med..

[47] Awan M.S., Irfan B., Zahid I., Mirza Y., Ali S.A. (2017). Comparison of Polymerase Chain Reaction and Immunohistochemistry Assays for Analysing Human Papillomavirus Infection in Oral Squamous Cell Carcinoma. J. Clin. Diagn. Res..

[48] Suárez-Lledó M., Marcos M., Cuatrecasas M., Bombi J.A., Fernández-Avilés F., Magnano L., Martínez-Cibrián N., Llobet N., Rosiñol L., Gutiérrez-García G. (2019). Quantitative PCR Is Faster, More Objective, and More Reliable Than Immunohistochemistry for the Diagnosis of Cytomegalovirus Gastrointestinal Disease in Allogeneic Stem Cell Transplantation. Biol. Blood Marrow Transplant..

[49] Raj G.D., Jones R.C. (1996). Immunopathogenesis of infection in SPF chicks and commercial broiler chickens of a variant infectious bronchitis virus of economic importance. Avian Pathol..

[50] van Ginkel F.W., Padgett J., Martinez-Romero G., Miller M.S., Joiner K.S., Gulley S.L. (2015). Age-dependent immune responses and immune protection after avian coronavirus vaccination. Vaccine.

[51] Gonzales-Viera O., Crossley B., Carvallo-Chaigneau F.R., Blair E.R., Rejmanek D., Erdoǧan-Bamac Ő., Sverlow K., Figueroa A., Gallardo R.A., Mete A. (2021). Infectious Bronchitis Virus Prevalence, Characterization, and Strain Identification in California Backyard Chickens. Avian Dis..

[52] Farsang A., Bódi I., Fölker O., Minkó K., Benyeda Z., Bálint Á., Kiss A.L., Oláh I. (2018). Coronavirus infection retards the development of the cortico-medullary capillary network in the bursa of Fabricius of chicken. Acta Vet. Hung.

[53] Pei J., Briles W.E., Collisson E.W. (2003). Memory T cells protect chicks from acute infectious bronchitis virus infection. Virology.

[54] Kotani T., Wada S., Tsukamoto Y., Kuwamura M., Yamate J., Sakuma S. (2000). Kinetics of lymphocytic subsets in chicken tracheal lesions infected with infectious bronchitis virus. J. Vet. Med. Sci..

[55] Najimudeen S., Barboza-Solis C., Ali A., Buharideen S.M., Isham I.M., Hassan M.S.H., Ojkic D., Van Marle G., Cork S.C., van der Meer F. (2022). Pathogenesis and host responses in lungs and kidneys following Canadian 4/91 infectious bronchitis virus (IBV) infection in chickens. Virology.

