# Supplementary material for: Comparison of Infectious Bronchitis Virus (IBV) Pathogenesis and Host Responses in Young Male and Female Chickens
Source: Viruses. 2023 Nov 22;15(12):2285. doi: 10.3390/v15122285 (PMC10747771; doi:10.3390/v15122285)
Supplement: Supplementary file 1 [file viruses-15-02285-s001.zip › viruses-2713912-supplementary.pdf]

Supplementary Materials:

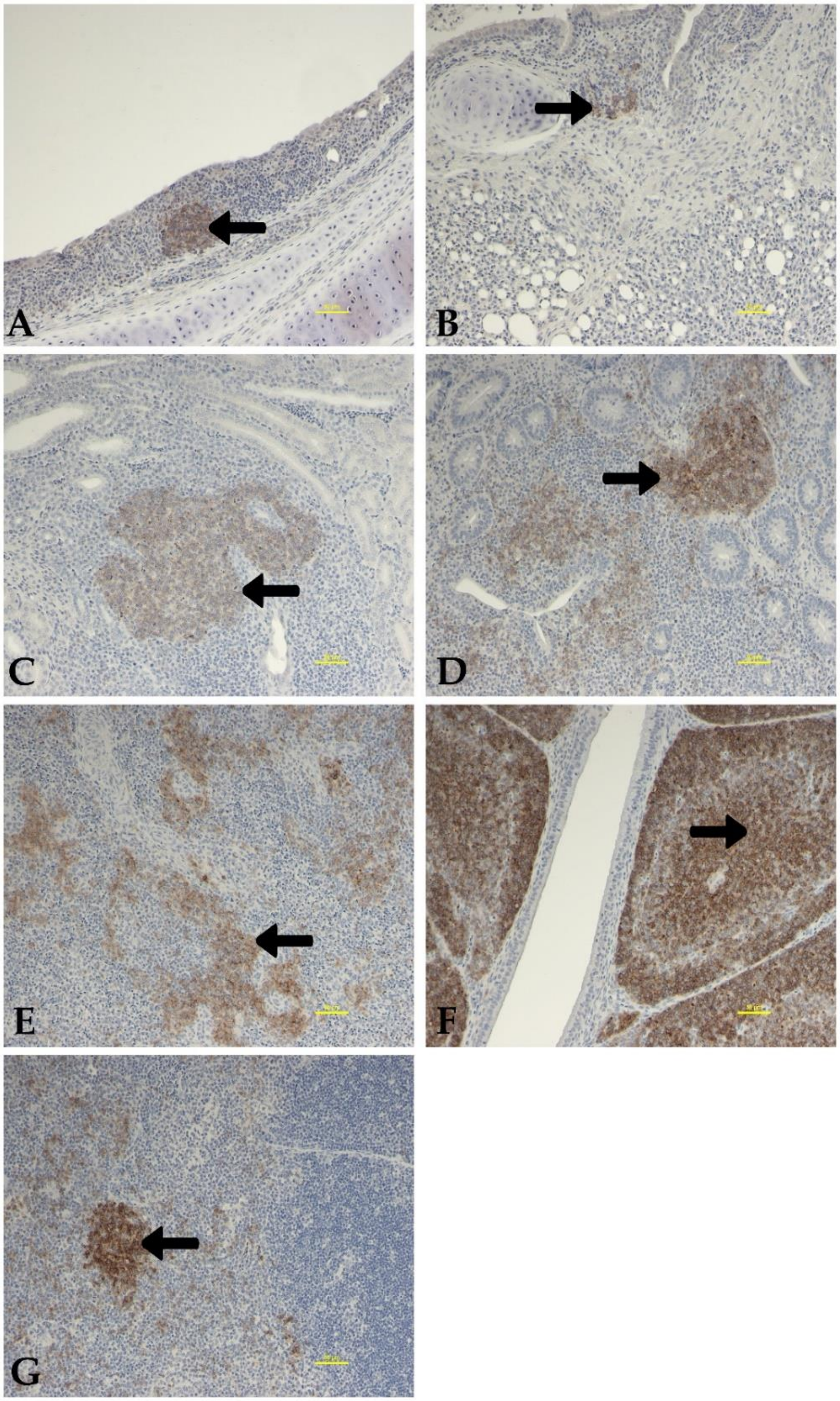

Figure S1. Immunohistochemical detection of B (Bu-1+) lymphocytes in trachea (A), lung (B), kidney (C), cecal tonsils (D), spleen (E), bursa of Fabricius (f), and thymus (G) collected at 4 and 11 dpi following infection with Canadian IBV DMV/1639 strain. Bu-1+ cells were stained with deep brown (indicated with black arrows). Scale bar = 50 μm.

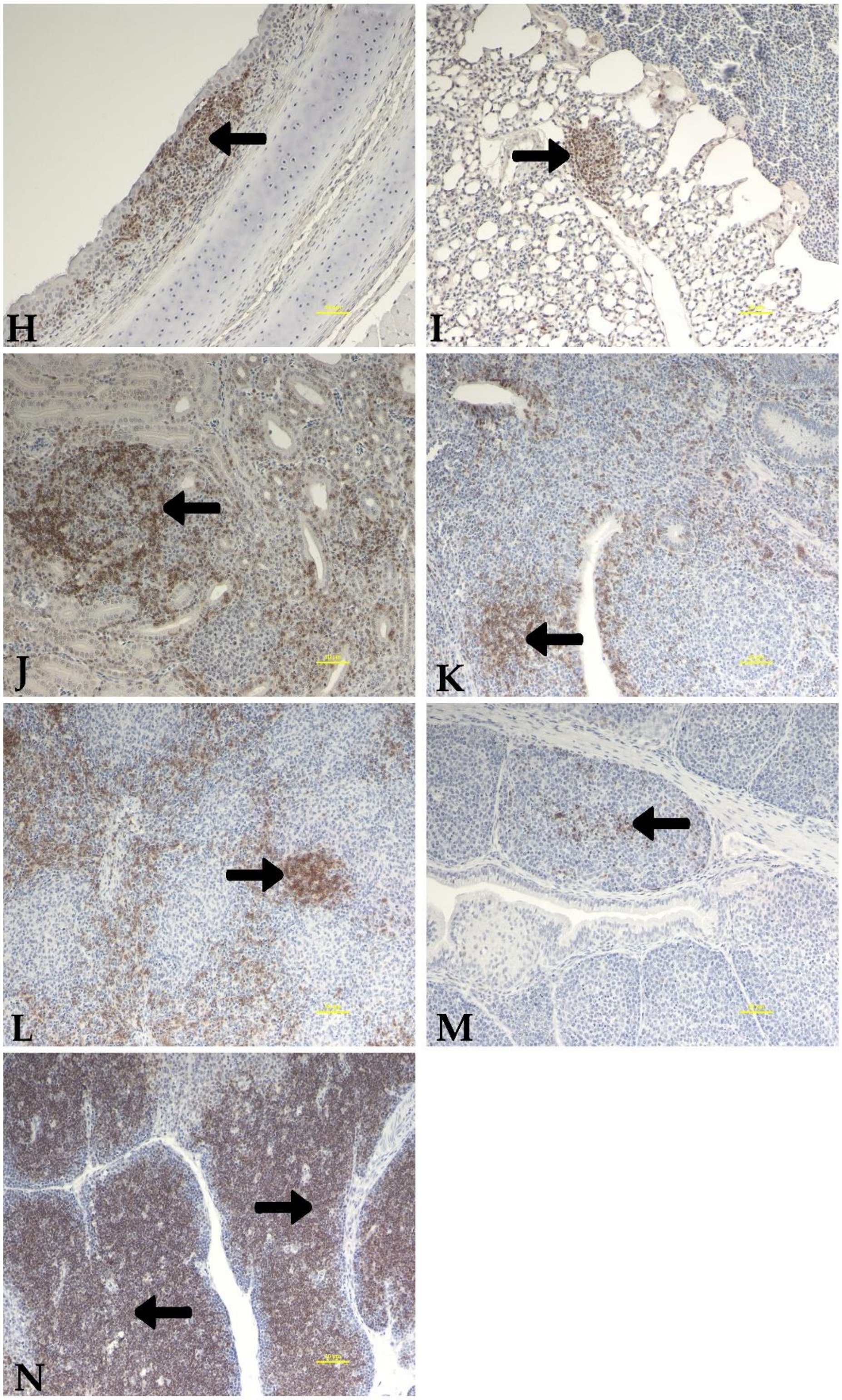

Figure S2. Immunohistochemical detection of CD8+ cells in the trachea (H), lung (I), kidney (J), cecal tonsils (K), spleen (L), bursa of Fabricius (M), and thymus (N) collected at 4 and 11 days following infection with Canadian IBV DMV/1639 strain. CD8+ cells were stained with deep brown (indicated with black arrows). Scale bar = 50 μm.
